# Treating People With Epilepsy in Rural Low-Income Countries Is Feasible. Observations and Reflections From a “Real Life Experience” After a Long Lasting Intervention in the Rural Chaco

**DOI:** 10.3389/fneur.2018.00855

**Published:** 2018-10-10

**Authors:** Alessandra Nicoletti, Loretta Giuliano, Chiara Colli, Calogero Edoardo Cicero, Sandra Padilla, Estela Vilte, David Rojo Mayaregua, Maria Del Carmen Martinez, Mario Camargo, Mario Zappia, Alessandro Bartoloni, Elizabeth Blanca Crespo Gómez

**Affiliations:** ^1^Department of Medical and Surgical Sciences and Advanced Technologies “G. F. Ingrassia, ” Section of Neurosciences, University of Catania, Catania, Italy; ^2^Department of Experimental and Clinical Medicine, Infectious Diseases Unit, University of Florence, Florence, Italy; ^3^Center of Anthropological Researches of the Teko Guaraní, Gutierrez, Bolivia; ^4^Bolivian League Against Epilepsy, Santa Cruz, Bolivia; ^5^Hospital Universitario Hernández Vera, Santa Cruz, Bolivia

**Keywords:** epilepsy, treatment, low-income countries, management, real-life

## Abstract

**Introduction:** Epilepsy represents an important public health issue, in particular in low and middle-income countries where significant disparities are present in the care available for patients with epilepsy. Treatment cost and unavailability of drugs represent important barriers in treating people with epilepsy especially in rural setting. Aim of the study was to evaluate, by means of routine data, the current real-life clinical practice in epilepsy in the rural communities of the Plurinational State of Bolivia. Treatment activity followed educational campaigns and an anthropological fieldwork over more than 20 years.

**Material and Methods:** Medical records of people with epilepsy (PWE) living in the rural communities of the Bolivian Chaco who received antiepileptic drugs (AEDs), from 2012 to 2016, and were followed-up for at least 1 year were analyzed. Treatment delivery and follow up visits were managed by a neurologist with the support of rural health care workers.

**Results:** From 2012 to 2016, 157 PWE (76 men with a mean age of 24.2 ± 15.7) have been included in the study. Structural epilepsy was the most common type, recorded in 54 cases (34.4%) and the most common reported causes were perinatal factors, present in 11 subjects (20.0%). Almost all patients presented epilepsy with generalized tonic-clonic seizures (91.4%). The most common AED prescribed was phenobarbital followed by carbamazepine. During the follow-up, a dramatic seizures reduction was observed, with 31 subjects (19.7%) being seizures-free at the last follow-up. However, 48 subjects (30.6%) did not assume the medication regularly and 10 interrupted the drug intake. More than 20% of PWE did not receive any financial supports for AEDs. During the follow-up period 10 patients died but only in one case the death was probably caused by epilepsy.

**Conclusion:** Our study demonstrated that PWE in rural areas of the Bolivian Chaco are willing to seek medical attention and to receive antiepileptic treatment. However, improvement in care is needed to assure compliance to AED treatment, including activity to increase awareness toward epilepsy among community members and health staff of the rural communities and to guarantee the coverage of treatment costs and drug supply.

## Introduction

Throughout the world, epilepsy represents an important public health issue, accounting for an estimated 0.7% of the global burden of diseases. It affects approximately 70 million people worldwide of whom the majority live in low and middle-income countries (LMIC) (1). In particular, about 5 million people living in Latin American Countries (LAC) are affected by epilepsy (2). In the Chaco region, which is part of the Plurinational State of Bolivia, it was found a prevalence of lifetime epilepsy of 12.3/1,000 and a prevalence of active epilepsy of 11.1/1,000. In a recent study the life-time prevalence of epilepsy associated with generalized tonic-clonic seizures (GTCS) was found to be 7.2/1,000 and the prevalence of its active form 6.6/1,000 with a crude incidence risk of 55.4/100,000 in the same area (3–5). According to a recent meta-analysis in Latin American countries the treatment gap (TG) is 60.6% (95% CI 45.3–74.9), with high differences between rural (77.8%; 95% CI 67.4–86.8) and urban (26.2%; 95% CI 10.2–46.4) areas (5). In LMIC there are significant disparities in the care available for patients with epilepsy (PWE). Most neurologists work in the urban private sector, where the level of care is similar to that found in developed countries. However, in the poorer urban and rural areas this level of care is rarely available (6). In this latter setting, indeed, general practitioners (GPs) have an important role in providing care and support to PWE. However, in rural areas non-medical health workers such as nurses and community health workers (CHWs) are often the only health care staff present who can diagnose epilepsy with GTCS. As pointed out by recent WHO recommendations, in LMIC settings epilepsy with GTCS, should be recognized and treated at primary care level by trained non-specialist health care providers (7).

Thus, education of primary health workers and other clinicians working in the rural areas represents a key action in the reduction of the treatment gap. Nonetheless treatment cost and unavailability of drugs represent a further important barrier in treating PWE in rural setting (6). Consequently, specific programs for the improvement of knowledge and awareness about epilepsy in these settings are not sufficient if not accompanied by governmental actions aimed to support the treatment cost and drug supply. The reduction of epilepsy TG in the area of Chaco has been the main aim of many different projects performed by our group over the last 20 years in this area (3, 4, 8–12). However, even if a reduction of TG was recorded over the time by the subsequent studies carried-out (3, 4, 13), the real impact of our activity in the clinical practice of PWE has never been evaluated until now.

Aim of the present study was to evaluate, by means of routine data coming from the rural communities of the Bolivian Chaco, the current real-life clinical practice in epilepsy in the rural communities of the Plurinational State of Bolivia.

## Materials and methods

### Study area

The study has been conducted in the Chaco area of the Plurinational State of Bolivia, which is a low-middle income country where about 4 million people live under the “poverty line” (14). In particular in rural areas the access to the health system is still difficult (15). However, a slight economic improvement has been recorded in the last few years, since the approval of the 2016-2020 National Economic and Social Development Plan by the Government of Bolivia aimed at maintaining growth of 5% and reducing poverty (16, 17). Indeed, with the application of policies to improve the economy, the social and health services and thanks to specific programs such as Bono Juana Azurduy, Programa Mi Salud, Ley de Gratuidad, Seguros departamentales, there was an increase in the social security and an improvement of the Bolivian health system (18).

**Figure 1:**
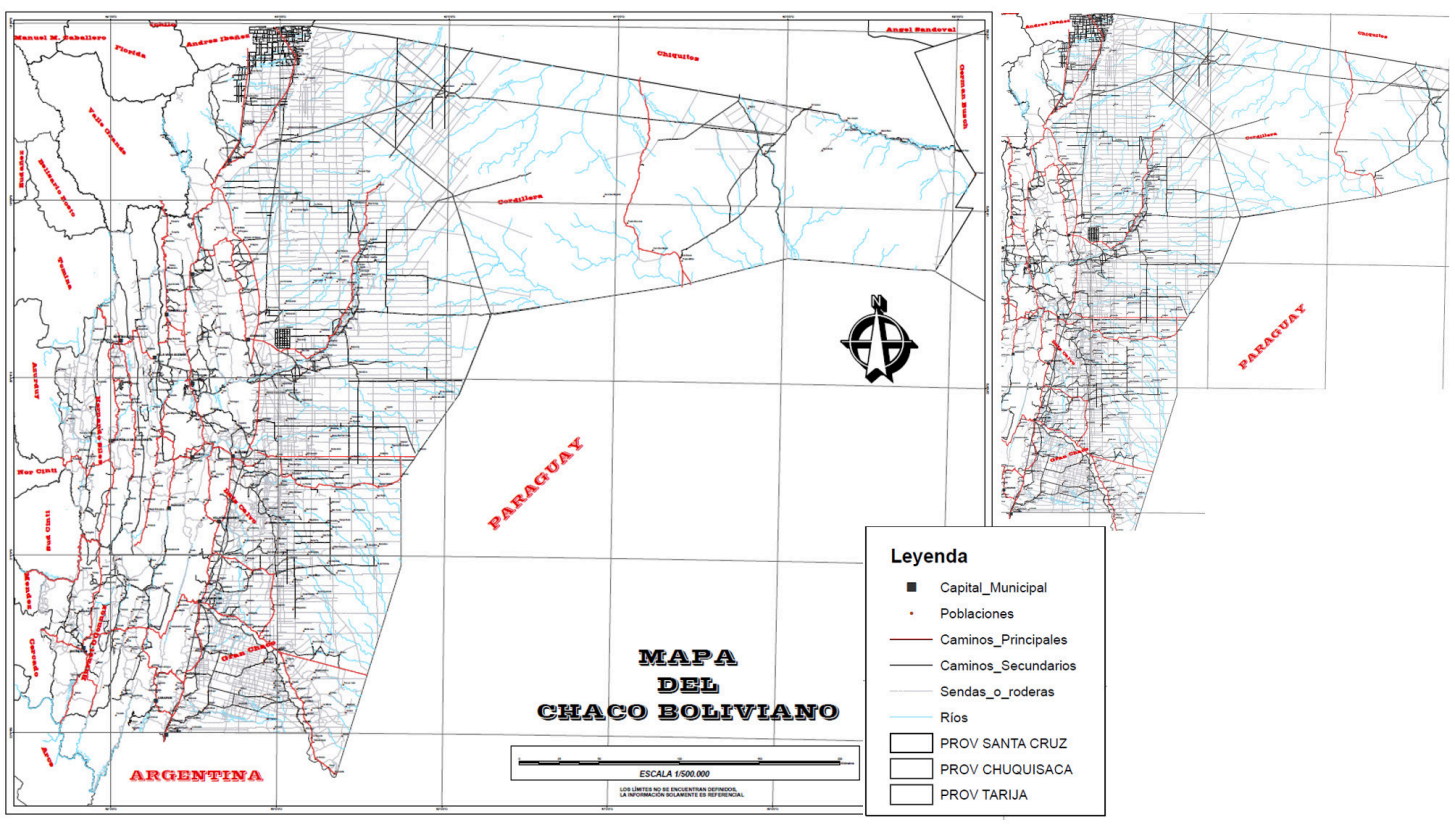
Map of the Plurinational State of Bolivia with highlighted on the top left the area of the Chaco region and the three departments of Santa Cruz, Chuquisaca and Tarija.

PWE involved in this study come from three Departments of the Plurinatinal State of Bolivia: the Department of Santa Cruz (municipalities of Boyuibe, Cabezas, Cuevo, Lagunillas, Gutierrez, Eiti, Camiri and S. Antonio Parapetí), the Department of Chuquisaca (municipalities of Macharetí and Muyupampa or Villa Vaca Guzmán) and the Department of Tarija (municipality of Villamontes). These Departments are part of the Chaco region (Figure 1), that is a subtropical area, inhabited mainly by indigenous Guaraní people. They live in communities that often lack basic services such as running water or electricity, basing their economy on animal husbandry and agriculture.

### Study population

About 190,000 people live in the Bolivian Chaco and among them about 80,000 are indigenous Guarani. It is estimated that 49% of the population live in rural areas with 68.9% of the population living in conditions of poverty, the majority of them still presenting unresolved issues such as energy and sanitation supplies^1^. In the last 25 years, many activities aimed at improving knowledge about epilepsy and reducing TG have been conducted by our group in the rural communities of the Chaco region. (8–14). After the assessment of the prevalence, incidence, mortality and the most frequent causes of epilepsy, the evaluation of the sociocultural dimension of epilepsy and epilepsy-associated stigma among members of the Guaraní communities, many training courses about epilepsy have been organized directed to the health staff and all the members of the communities (3, 4, 8–13, 19). In particular an educational campaign directed to GPs and non-medical health staff has recently been implemented with teaching courses regarding the main causes of epilepsy, epilepsy diagnosis and treatments, first aid, prevention of the secondary forms of epilepsy and psychosocial aspects such as social stigma and discrimination (10, 19).

From 2012 to 2016 a Bolivian neurologist, working in Santa Cruz, participated to the teaching activity, organized in collaboration with the “Convenio de Salud” and the “Escuela Tekove Katu.” The “Convenio de Salud,” located in Camiri, works through cooperation projects with the University of Florence in Italy and the Pan American Health Organization. Its objective is to improve the health conditions of the indigenous Guaraní population through the development of health projects in primary care. The “Tekove Katu” school, located in Gutierrez, carries out training courses since 1985 and is an integral part of the educational and health system of the Plurinational State of Bolivia^2^. The neurologist guaranteed a bimonthly service of neurological visits to PWE living in the rural communities of the Chaco region.

Subjects identified by trained nurses or CHWs, as well as PWE already diagnosed by the GPs working in the rural areas or identified during the different epidemiological surveys, were attended in the main health centers located in the different areas.

When the diagnosis of epilepsy was confirmed, AEDs were prescribed and for PWE already in AED treatment, prescription was confirmed according to the neurologist's judgement.

This clinical activity was not part of a specific scientific programme and clinical data were routinely collected without the aim to be analyzed. Consequently, due to the lack of a standardized data collection, often incomplete information was gathered with a certain amount of missing data.

**Figure 2:**
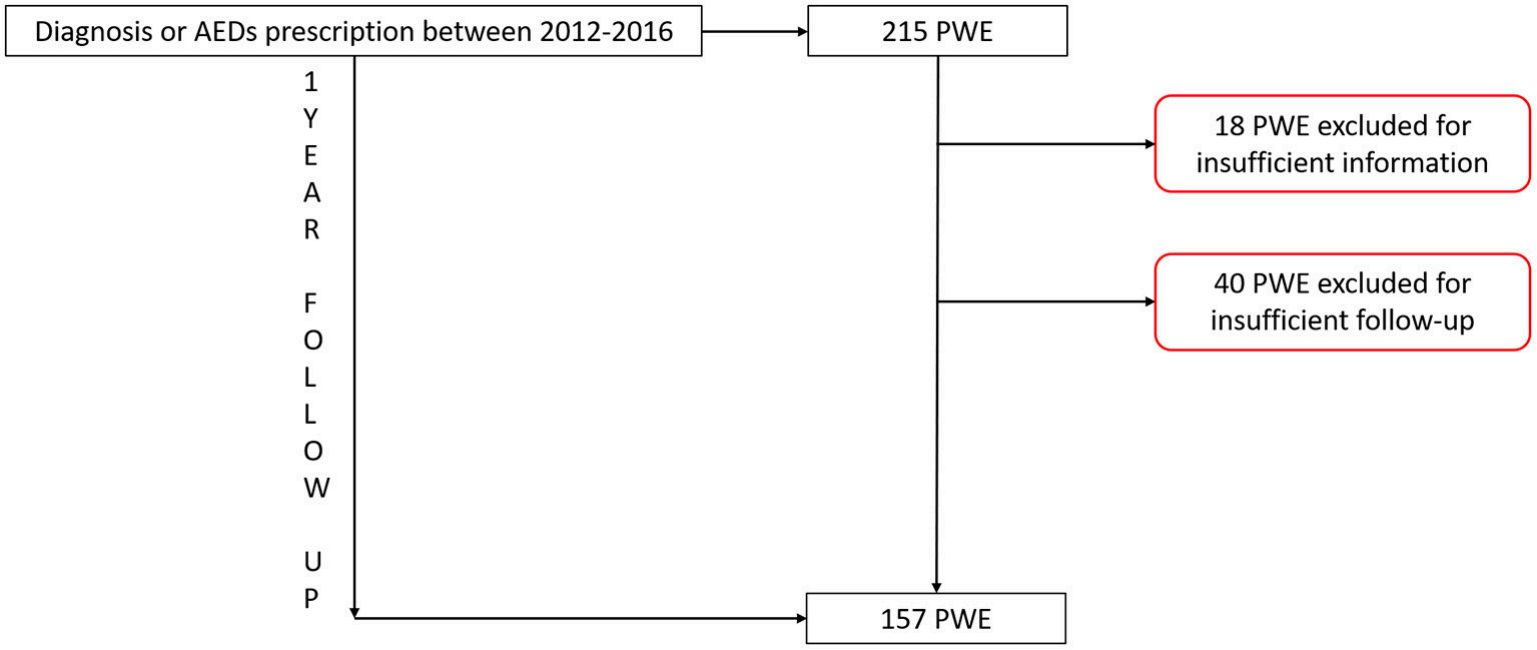
Flow chart describing the study population. AEDs, antiepileptic drugs; PWE, people with epilepsy.

All medical records of all PWE coming from rural communities who received AEDs treatment and were followed-up for at least 1 year were analyzed by two independent observers (LG, CEC). Those patients for whom not sufficient information was available or who underwent only one neurological visit without follow-up have been excluded (Figure 2).

Diagnosis of epilepsy was generally performed just on the bases of the clinical history because the majority of cases never underwent EEG recording or neuroimages. On the bases of the clinical history, epilepsy was defined according to the ILAE definition and classification proposed in 2017 (20). In particular, according to the causes, epilepsy was classified as “structural” when an imaging abnormality was the likely cause of the patient's seizures; “genetic generalized” when an idiopathic form with a genetic predisposition was suspected on the bases of the clinical history; “of unknown etiology” when the cause of the epilepsy was not yet known; “unknown” when there was not adequate information to classify epilepsy type into a category (20).

Among the causes of structural epilepsy, perinatal factors were defined when there was an evidence of a hypoxic-ischemic encephalopathy (21); traumatic brain injury was defined as an insult to the brain from an external mechanical force, with an associated diminished or altered state of consciousness (22); neurocysticercosis (NCC) was defined according the most recent criteria (23); for the other possible causes we based our diagnosis on the previous clinical report.

### Data management and statistical analysis

Medical records were revised twice by two different operators (CC, LG) and relevant data were collected in an *ad hoc* created standardized form. Data were double entered into the database and statistical analysis was performed using the software STATA 12 (version 12.0, College Station, TX). Data cleaning was performed before the data analysis considering range and consistency checks. Quantitative variables were described as mean and standard deviation (SD) while frequencies using percentages. The frequency comparisons were performed using the chi-squared test.

### Ethical approval

The study has been approved by the Ethical committee of the Bolivian Neurological Society.

## Results

From 2012 to 2016, 215 PWE from the rural communities of the Gran Chaco area underwent a neurological examination and received at least an AED prescription. Of these, 18 were excluded from the analysis because no sufficient information was available, while 40 were excluded due to the lack of a follow-up visit. Finally, 157 PWE (76 men with a mean age of 24.2 ± 15.7) who had had at least 1 year of follow up were included in the analysis. The majority of them came from the areas of Camiri, Gutierrez and Eiti as shown in Table 1. Baseline characteristics are shown in Table 1.

**Table 1:** Demographic features of the patients.

	***N* = 157 (%)**
Sex (M)	76 (48.4)
Mean age	24.2 ± 15.7
Mean age at onset	9.6 ± 12.2
**OCCUPATION**	
Unemployed	30 (19.1)
Student	39 (24.8)
Housewife	29 (18.5)
Farmer	15 (9.5)
Other	10 (6.4)
Not known	34 (21.7)
**AREA OF ORIGIN**	
Boyuibe	12 (7.6)
Cabezas	13 (8.3)
Camiri	45 (28.7)
Cuevo	4 (2.5)
Eiti	30 (19.1)
Gutierrez	25 (15.9)
Lagunillas	9 (5.7)
Macharetí	9 (5.7)
Muyupampa	2 (1.3)
San Antonio Parapetí	8 (5.1)

*N, number; M, men*.

The mean age at epilepsy onset was 9.6 ± 12.2. Seizure types were classified on the bases of the clinical history which was available in 113 cases (72%). Almost all patients presented epilepsy with GTCS of whom 26 (23.0%) focal to bilateral tonic-clonic seizures, while 81 (71.7%) were classified as generalized tonic-clonic. Only 6 (5.3%) presented focal seizures (Table 2). Structural epilepsy was the most common type of epilepsy, recorded in 54 cases (34.4%) followed by epilepsy of unknown etiology (22 cases, 14.0%) and genetic generalized epilepsy (21, 13.4%). Nonetheless, for 60 cases (38.2%) epilepsy was defined as unknown (Table 2).

**Table 2:** Classification and causes of epilepsy.

	***N (%)***
**CLASSIFICATION OF EPILEPSY**	
Focal epilepsy	76 (48.4)
Generalized epilepsy	21 (13.4)
Unknown epilepsy	60 (38.2)
**CLASSIFICATION OF EPILEPSY BASED ON THE ETIOLOGY**	
Structural epilepsy	54 (34.4)
Genetic generalized epilepsy	21 (13.4)
Epilepsy of unknown etiology	22 (14.0)
Unknown epilepsy	60 (38.2)
**CLASSIFICATION OF SEIZURES (*****N** =* **113)**	
Focal	6 (5.3)
Focal to bilateral tonic-clonic	26 (23.0)
Generalized tonic-clonic	81 (71.7)

*N, number*.

Concerning the causes of structural epilepsy, perinatal factors were the most commonly reported, being present in 11 subjects (20.4%), followed by head trauma (11.1%). A diagnosis of meningitis was reported in 5 cases, NCC in 3 while other structural causes such as stroke or cysts were reported in 6 cases. For 22 cases (14.0%) “known” causes were not reported and were then classified as epilepsy of unknown etiology. Clinical characteristics of epilepsy are shown in Table 2.

Presence of co-morbidities was reported in 24 cases (15.3%) and the majority (17 subjects; 10.8%) presented developmental delay; however, for 22 cases this information was missing.

**Figure 3:**
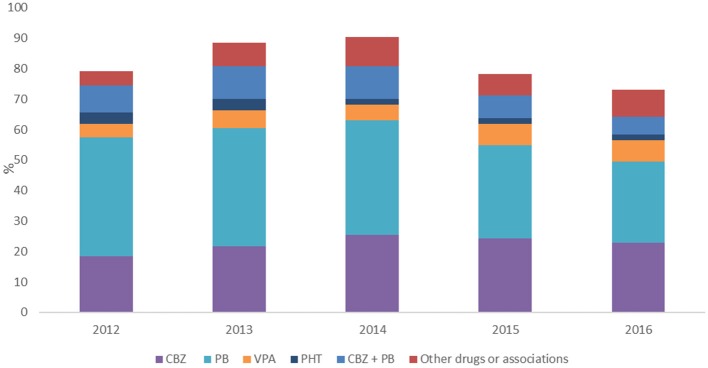
Percentage of subjects treated with different AEDs per year. CBZ, carbamazepine; PB, phenobarbital; VPA, valproic acid; PHT, phenytoin.

Overall, the most common AED prescribed was phenobarbital followed by carbamazepine and by the association of phenobarbital and carbamazepine. AEDs treatment during the follow-up period (2012–2016) is reported in Figure 3.

**Figure 4:**
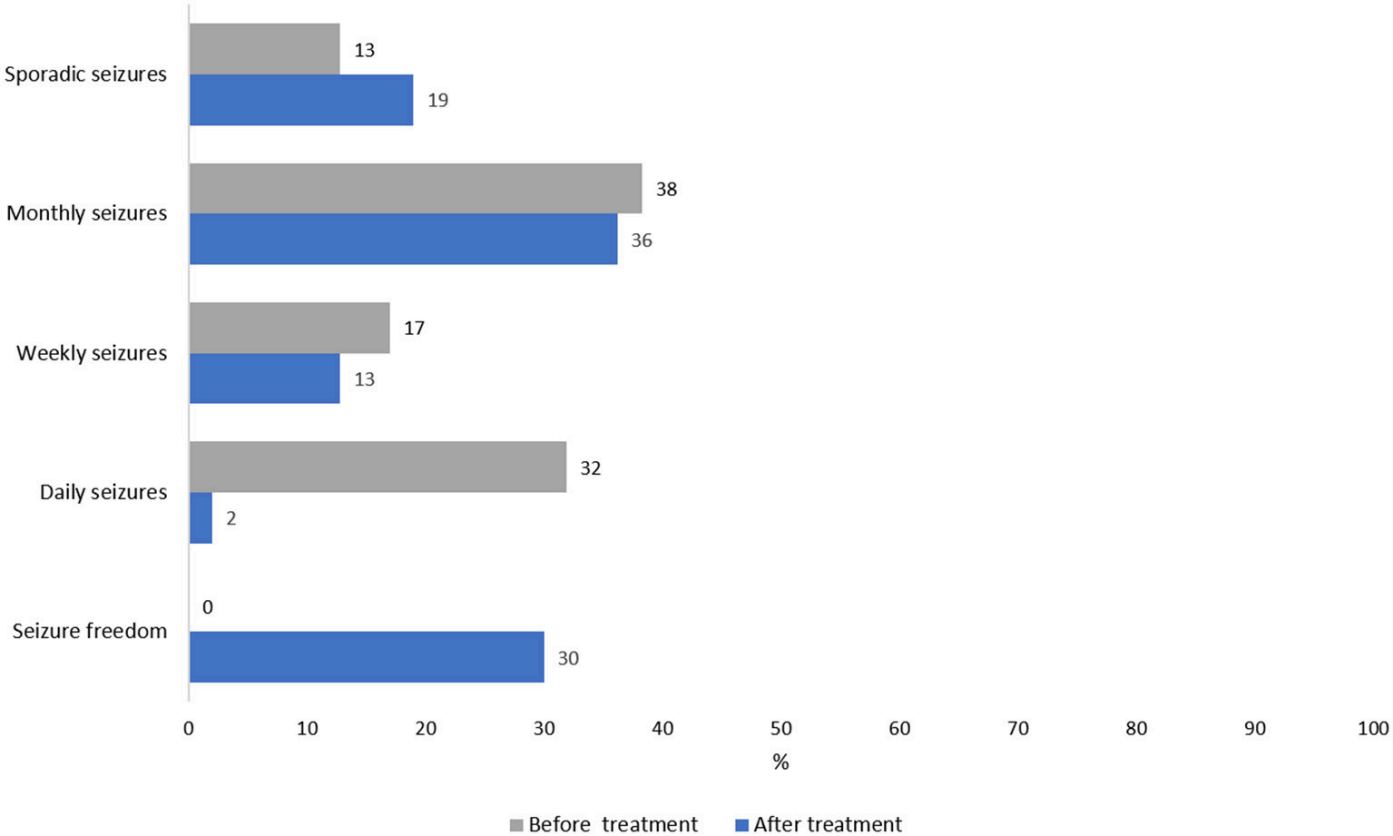
Frequency of seizures before and after treatment in 47 patients with epilepsy.

Information about seizure frequency before and after the treatment were available only for 47 patients. Before the AEDs treatment only 13% of cases presented sporadic seizures (2–3 per year) while the majority (55%) presented monthly or weekly seizures and 32% reported to have seizures every day. During the follow-up, a dramatic seizures reduction was observed and, in particular, 14/47 (30%) were seizure-free during the last year of follow-up, while 19% presented only sporadic seizures, and most of them just when they did not take the AEDs regularly. Frequency of seizures before and after treatment for these patients is reported in Figure 4. Of 157 PWE, 31 (19.7) were seizure-free at the last follow-up.

It should be underlined that 48 subjects (30.6%) stated to not assume regularly the medication and that 10 (6.4%) interrupted the drug intake. Of these, 8 (13.8%) interrupted AED treatment due to the absence of seizures, while for the others the most common reason for an irregular intake or interruption was the lack of medication, in part due to economic reasons. The cost of AEDs, in fact, was covered by the Ministry of Health programmes (Mi Salud and Seguro Universal Materno Infantil, SUMI) or by the municipality (“alcaldia”) for 21 (13.4%) patients, by the local Strategic Partner Organizations (SPOs) of the Non-governmental Organization (NGO) “Liliane Fonds” (20.4%) and by the Convenio de Salud (11.5%). At any rate, more than 20% of PWE did not receive any financial supports.

During the follow-up period, 10 (6.4%), of the 157 PWE, died. Of them, 9 died due to causes not directly related to seizures, while only in one case a seizure-related cause was identified. In particular, the causes of death were the followings: severe malnutrition in four cases, malnutrition and pneumonia in two cases, pneumonia in one case, alcohol addiction complications in two cases, severe burn following a fall during a seizure in another case. Five of the 10 subjects suffered from structural epilepsy and six (60%) had also other comorbidities. Among these subjects, three had suspended the treatment, while five declared an irregular intake. The patient for whom a seizure-related death has been suspected was suffering from epilepsy with GTCS and alcoholism and he had suspended his treatment because of alcohol abuse.

## Discussion

Epilepsy treatment gap is an important public health problem in LMICs and in particular in the rural areas. Inadequate trained staff, cost of antiepileptic treatment and its unavailability, cultural misbeliefs about epilepsy, use of traditional medicine, and distance from the health posts represent its main causes (6) and appropriate interventions have been suggested to reduce it (24).

Among the suggested interventions, the core actions are represented by training nurses and community health workers, in order to be able to identify people with epilepsy, and training GPs working in the rural communities in order to diagnose and initiate epilepsy treatment (10). Indeed, according to the WHO guidelines, in LMICs settings epilepsy with GTCS should be diagnosed at primary care level by trained non-specialist health care providers (7). Moreover, increasing communities' awareness about epilepsy represents a further important action in order to reduce stigma and improve the adherence to the epilepsy treatment, even if misconceptions about epilepsy and recourse to traditional healers are challenging to change.

During our long-lasting activity in rural Bolivia, in agreement with the ILAE and WHO recommendations, from 1994 up to date we performed several epidemiological and interventional surveys including an anthropological survey, training programs directed to GPs, nurses and CHWs of the rural communities of the Chaco region as well as communities awareness programs (ongoing activity). Training activities were mainly directed to the Knowledge, Attitudes and Practice (KAP) and management of epilepsy with GTCS. The activities conducted by our group during these last 25 years, have surely increased the level of knowledge and awareness about epilepsy in this region as demonstrated by the drop in the level of treatment gap recorded during different epidemiological survey over the time. Indeed, in our first survey performed in 1994 a treatment gap of about 90% was recorded while in 2010 a reduction of TG to values of about 70% has been found; finally in 2016 a value of TG of 45% has been recorded (3, 4, 13).

Although recognizing the importance of data coming from well-designed epidemiological studies, we believe that it can be worthwhile to evaluate the impact of our activity on the “real life” and not only under experimental conditions. Several trials have already demonstrated the feasibility of the treatment of epilepsy with GTCS under experimental conditions (25), but few data are available regarding the following step, namely the clinical practice in these settings. In experimental conditions, in fact, PWE are strictly monitored, the treatment is guaranteed and often delivered directly in the communities; furthermore, sometimes the costs of the AEDs have been directly covered by the project. However, what happens after the trial? Which is the real adherence to the treatment and the risk of an abrupt interruption? Which are the main causes of an irregular intake? All these important questions can be addressed only by the analysis of routine data, taking into account all the limits related to this kind of study. In fact, routine data collected for the clinical practice have the fault of lacking information with possible missing data for many subjects and many variables. Moreover, we are absolutely aware about the possible selection bias related to the fact that PWE treated and included in the analysis can have different characteristics with respect to those never treated. Nonetheless, from our point of view, despite all the possible limits, important food for thought comes from this study.

As expected on the bases of our activities that, in agreement with the WHO guidelines, have been focused on the management of GTCS, more than 90% of PWE on treatment presented convulsive epilepsy. Concerning the epilepsy causes, it was possible to define the epilepsy type only in a minority of cases, since in 14% of cases epilepsy was defined as of unknown etiology and in 38.2% of patients, epilepsy diagnosis was not possible. This high level of diagnostic uncertainty, with consequent underestimation of the most frequent recognized epilepsy causes, such as NCC (11), is certainly due to the low availability of reliable diagnostic tools in these areas. Treating epilepsy with GTCS in rural communities, regardless of its causes, is feasible as demonstrated by the high frequency of a regular intake and the dramatic seizures reduction. Nonetheless 30% of subjects reported an irregular intake often due to irregular AEDs supply and economic reasons. On the other hand, AEDs adherence does not seem to be influenced by stigma or cultural beliefs. Thus, when we increase knowledge and awareness about epilepsy, the treatment is well accepted and sought by PWE. However, cost and supply still represent an important barrier in these areas. Currently, the costs of AEDs are in part covered by NGOs or other non-governmental institutions. An effort should be made to extend the cost coverage of AEDs by national and departmental health institutions since the cost of the most common AEDs used in Bolivia is very low. In fact, considering the most common drugs used, the annual cost of a treatment with the average dosage used of 100 mg of PB daily would be only about 50 $ per person a year, as well as the annual treatment with 400 mg of CBZ, which would cost about 95 $ per person a year. Only 21.6% of cases are currently taken in charge by the municipality and Ministry of Health (Mi Salud and SUMI). Furthermore, the follow-up depends entirely by the volunteer action of local neurologists. It should be underlined that even if the follow-up can be performed by the GPs working in the rural area, in Bolivia, as well as in other countries, phenobarbital, that represents the first line drug, can be prescribed only by neurologists. In this perspective, the presence of a neurologist should be considered as an essential element within a team attending PWE, acting as prescriber and reference specialist for GPs. Programs should consider this presence and the relative costs.

Mortality rate for epilepsy in the Chaco area was 10/1,000 (12) and even if, due to the different study design, a direct comparison cannot be made, the number of deaths recorded in this sample (10 out of 157 PWE) is probably higher than expected. As already reported it is possible that patients seeking treatment and consequently included in the study were the most severe ones, thus leading to a possible selection bias. Indeed, patients who died were affected by more severe forms of disease as demonstrated by a high rate of comorbidities present in 60% of subjects, mainly represented by developmental delay. Moreover, all patients presented a structural epilepsy with only GTCS and with a frequency higher with respect to the rest of the sample (57.15% with weekly or daily seizures before treatment). In agreement with this observation, nine patients died due to causes not directly related to seizures, such as severe malnutrition, pneumonia and alcohol addiction, while only in one case a seizure-related cause was identified (severe burn following a fall during a seizure). Nonetheless, this latter patient interrupted his treatment intake because of alcohol abuse.

We are aware that our study includes only a part of PWE residing in this area. Indeed, considering the total rural population of the study areas (about 75,000 inhabitants according to the 2012 census) and according to the prevalence of active epilepsy associated with GTCS (6.6/1,000) (4) which is the most common type, about 495 PWE are expected in this population. Thus, we can conclude that about 50% of PWE (*n* = 215) received medical attention and were treated with an AED in this area, percentage that is roughly close to the last TG recorded in the same area (13). Nonetheless, this is not a population survey and, as reported, selection bias is probable. It is possible to hypothesize, in fact, that PWE affected by more severe forms were more prone to seek medical attention and treatment. To these reasons, our data do not allow to reach any conclusion regarding the effective TG. According to our experience, after improving knowledge and awareness in the rural population, PWE are willing to seek medical attention and to receive antiepileptic treatment, and these considerations can be extended to other similar settings.

At this point governmental and departmental actions are urgently needed in order to guarantee an adequate treatment and medical attention in these areas. In particular, local government should strengthen specific training programs for the GPs and CHWs living in the rural area and should support supply of generic, low-cost, first-line AEDs. These actions could lead to a significant improvement of epilepsy care in rural areas.

Furthermore, the national organizations and international official epilepsy institutions should reinforce their actions in order to obtain equal conditions people with epilepsy in the different parts of the world.

## Author contributions

LG and AN contributed to conception and design of the study. SP, EV, DR, and MM contributed to the organization of the project. LG, AN, CC, and CEC organized the database. LG and AN performed the statistical analysis. LG and AN wrote the first draft of the manuscript. CC, CEC, and AB wrote sections of the manuscript. MZ, MC, AB, and EC critically reviewed the manuscript. All authors contributed to manuscript revision, read, and approved the submitted version.

### Conflict of interest statement

The authors declare that the research was conducted in the absence of any commercial or financial relationships that could be construed as a potential conflict of interest.

## Footnotes

^1^*OPS/OMS Paraguay - Rostros, Voces y Lugares: El Chaco Sudamericano*. Available online at: https://www.paho.org/par/index.php?option=com_docman&view=document&layout=default&alias=139-rostros-voces-y-lugares-el-chaco-sudamericano&category_slug=salud-familiar-y-comunitaria&Itemid=253 (Accessed September 4, 2018).

^2^*Home - Scuola di Salute Pubblica Tekove Katu*. Available online at: http://www.tekovekatu.org/ (Accessed January 30, 2018).
